# Relationship between the number of hospital pharmacists and hospital pharmaceutical expenditure: a macro-level panel data model of fixed effects with individual and time

**DOI:** 10.1186/s12913-020-4907-2

**Published:** 2020-02-05

**Authors:** Ming Wei, Xuemei Wang, Dandan Zhang, Xinping Zhang

**Affiliations:** School of Medicine and Health Management, Tongji Medical College, Huazhong University of Science and Technology, Wuhan, Hubei China

**Keywords:** Hospital pharmacist, Pharmaceutical expenditure, Relationship, Panel data, Fixed effect model

## Abstract

**Background:**

The rapid increase in pharmaceutical expenditure (PE) has been a main problem of global healthcare reform for decades. Previous studies demonstrated that pharmacists play an indispensable role in controlling PE, but macro-research evidence is scarce. Exploring the role of pharmacists from a macro-perspective is essential for pharmacy source allocation with an advantage of extensive applicability over regions. This study aimed to explore the relationship between the number of hospital pharmacists and hospital PE and to provide a macro-perspective evidence to curb the increasing PE and decline unnecessary medications.

**Methods:**

Data were extracted from China Health Statistics Yearbook from 2011 to 2018. A panel dataset with 31 provinces from 2010 to 2017 was constructed. Amongst them, ‘Number of hospital pharmacists per 1 million of population’ (HLPT) was selected as an independent variable, ‘Per visit of hospital outpatient pharmaceutical expenditure’ (OTPE) and ‘Per capita of hospital inpatient pharmaceutical expenditure’ (ITPE) were selected as dependent variables, and ‘Number of hospital physicians per 1 million of population’ (HLPN) and ‘Drug price index’ (DPI) were applied as control variables. Fixed-effect panel data analysis was performed to evaluate the relationship between the number of hospital pharmacists and hospital PE.

**Results:**

HLPT had a significant and negative relationships with OTPE (β_1_ = − 0.0893, *p* = 0.0132) and ITPE (β_1_ = − 4.924, *p* < 0.001). Considering the control variables, the significant and negative relationships with HLPT and OTPE remained unchanged (β_1_ = − 0.141, *p* < 0.001; β_1_ = − 4.771, *p* < 0.001, respectively), indicating that an increase in hospital pharmacist per 1 million of population led to a decrease of ¥474 million ($67.4 million) OTPE and ¥902 million ($128 million) ITPE in 2017. Overall, in 2017, an increase of 1 hospital pharmacist led to a decrease of approximately ¥1 million ($142 thousands) hospital PE nationwide.

**Conclusion:**

This study confirmed the negative relationship between hospital pharmacists and hospital PE, indicating that hospital pharmacists might play a significant role in controlling PE. Pharmacists were encouraged to participate in more drug-therapy-related activities, such as medication reconciliation.

## Background

Increasing pharmaceutical expenditure (PE) has been regarded as the main concern worldwide, and the global PE reached 1.2 trillion in 2018 [1]. As a key contributor to health expenditure growth, the sustainable growth of PE has become an important primary issue of China’s health reform [2].

Many countries have introduced policies to promote a pharmacist’s role in reducing PE. For instance, pharmacists in Germany, Denmark, the United States and the Netherlands are allowed to substitute generic drugs for proprietary brands [3]. In Germany, if a physician’s prescription level is more than 15% above the average, they will receive a reminder from pharmaceutical advisers to discuss their prescriptions [4]. In Washington State, pharmacists need to sign health plans to provide post-diagnosis medication management [5]. Previous researches have proved that pharmacists can reduce PE [6–8]. Tareq et al. [6] found that a clinical pharmacist’s intervention in ICU totally saves $211,574.90 of drug therapy cost through a comparative study. A randomised controlled trial has shown that pharmacists reviewing repeat prescriptions can reduce PE to £61 per elderly patient (≥65) per year [7]. Vazin et al. [8] found that clinical pharmacists help decline PE by 50.76% by controlling the use of three high-cost medications, including albumin, intravenous (IV) pantoprazole and IV immune globulin. Some studies have demonstrated that dispensing pharmacists can reduce drug and package costs by managing drug storage, logistics and other medicine-handling procedures [9], while most studies have drawn the conclusion that clinical pharmacists participate in a patient’s drug therapy, provide pharmaceutical care and reduce medication errors, which eventually cut down PE [10].

Previous studies mainly focused on exploring the role of pharmacists from a micro-perspective, and macro-perspective studies are few. Bond et al. [11] explored the relationship amongst clinical pharmacy services, pharmacist staffing and PE in hospitals in the United States through multiple regression analysis and showed that PE decreases as the number of clinical pharmacists increases(*p* = 0.018). They concluded that clinical pharmacists should be definitely more than 1.11/100 occupied beds [12].

Besides, a few macro-perspective studies have evaluated the relationship between the number of physicians and health expenditure (HE). Alihussein et al. [13] found that an increase in the number of physicians causes an increase in HE in Economic Cooperation Organisation countries. Livio [14] demonstrated that the addition of one physician per 1 million of population brings approximately 17 cents to per capita HE. Another study has shown that total HE increases by 1.21 standard deviations as the health workforce (including physicians, dentists, pharmacologists, nurses and midwives) growth rate increases by 1 standard deviation (*P* < 0.001, [15]. It can be seen that an increase in the number of physicians is related to an increase in HE, so an increase in the number of physicians might also be related to an increase in PE because PE is the main part of HE [16]. The abovementioned methods include fixed effect, pooled time-series cross-section regression and multiple linear regression based on panel data and time series data. Panel data analysis is considered for the ability to mitigate unobserved heterogeneity, which is quickly displacing their cross-sectional counterpart in many studies [17]. Fixed effects with individual and time obtain the heterogeneity estimation of entity and time parameters to improve the goodness of fit [18]. The control variables added to the model can diminish the effect of exogenous variables to achieve a more accurate estimation [19]. Given that some studies have explained the relationship between the numbers of physicians and HE using panel data and its advantages, we construct a panel data model to explore the relationship between the number of hospital pharmacists and hospital PE.

In 2008, PE accounted for as high as 42.10% of the total HE in China [20]. Subsequently, the figure decreased to 35.33% in 2017 after a series of regulatory policies was issued by the government [16]. However, the proportion is still higher than that in OCED countries, i.e. 14% on average [21]. Hu et al. [22] found that zero mark-up drug policy fosters overprescribing, although it decreases PE. Chinese researchers also studied the behaviour of clinical pharmacists controlling PE, such as prescription review [23], drug therapy participation [24] and pharmaceutical care providing [25], but all of them were analysed from a micro-perspective.

Given that macro-perspective evidence is scarce, it’s worth exploring whether the number of pharmacists influence PE in China [25]. Fixed effects were performed on the basis of a provincial panel data from 2010 to 2017 to investigate the relationship between the number of hospital pharmacists and hospital PE, and the influence of the number of physicians and Drug price index (DPI) was controlled simultaneously. This study aimed to investigate a hospital pharmacist’s role in reducing PE from a macro-perspective and provide evidence-based reference for clinical pharmacy development and hospital pharmacist allocation [17–19].

## Methods

### Data source and study variables

This retrospective study built a panel database containing 31 provinces from 2010 to 2017. ‘Number of hospital pharmacists per 1 million of population’ (HLPT) was selected as an independent variable, ‘Per visit of hospital outpatient pharmaceutical expenditure’ (OTPE) and ‘Per capita of hospital inpatient pharmaceutical expenditure’ (ITPE) were selected as dependent variables. ‘Number of hospital physicians per 1 million of population’ (HLPN) and DPI were added as control variables. The above variables are all provided in Additional file 1.

Because a hospital is one of the main workplaces of clinical pharmacists in China [26]. Government-stipulated secondary hospitals should allocate at least three clinical pharmacists and tertiary hospitals at least five in 2011 [27]. Hence, hospital pharmacist is typically used as a study object in our research owing to the data unavailable of clinical pharmacist. The regional number of hospital pharmacists and physicians was divided by the regional population, and HLPT and HLPN were obtained to reduce the deviation caused by regional and demographic differences. Because we chose hospital pharmacist as independent variable and many patients buy medicines directly from the hospital pharmacy after seeing doctors in hospital rather than go to other medical institutions or retail pharmacies [28], we used hospital PE as our dependent variable, which was constituted of outpatient PE and inpatient PE. Mousnad et al. [29] indicated that the main factors contributing to the increase in pharmaceutical expenditure are drug quantities, therapies and new drugs. Shi et al. [2] divided the determinants of pharmaceutical expenditure into the amount of prescriptions dispensed and price indices by using the consumer price index of medical care and pharmaceuticals and the proxy of health care utilisation as indicators, respectively. On the basis of these previous studies, we used DPI to measure price growth, and chose the number of physicians to control the factors of prescription growth, therapeutic change and new pharmaceutical products because physicians are the decision makers of medication use.

The data of hospital pharmacists, physicians and drug costs were extracted from China Health Statistics Yearbook of 2011 to 2018, which are the most authoritative databases reflecting the development of health care systems and citizens’ health status in China. Population data were extracted from China Statistics Yearbook of 2018, a database focusing on the development of the social economic status in China. The data of DPI were extracted from the website of the National Bureau of Statistics of China [30].

The specific calculation of two indicators (OTPE and ITPE) in our study were as follows:

(i) Per visit of hospital outpatient pharmaceutical expenditure (OTPE) = hospital outpatient drug sales/the number of outpatient visits [16].

(ii) Per capita of hospital inpatient pharmaceutical expenditure (ITPE) = hospital inpatient drug sales/the number of discharges [16].

Notes: Hospital outpatient (inpatient) drug sales are measured by the sum of the outpatient (inpatient) sales from traditional Chinese pharmaceutical medicine and Western pharmaceutical medicine in hospitals [16].

DPI was replaced with the Retail Price Index of Drugs and Medical Care Products due to the absence of annual regional data, and the index of 100 in 2010 was used to calculate the rest years.

Panel data model selection: fixed effect with both individual and time.

This study considered static panel analysis with likelihood and Hausman test to determine the final model.

Eviews 10.0 was used for empirical analysis. We used the likelihood and Hausman tests to determine the model of the number of hospital pharmacists and drug costs without control variables. Significant *P* < 0.01 indicated fixed effects with both individual and time as the appropriate model. Model 1 can be written as.

1 $$ otpeit=\alpha 1D1+\alpha 2D2+...+\alpha NDN+\gamma 1W1+\gamma 2W2+...+\gamma TWT+\beta 1 hlptit\hbox{'}+\varepsilon it $$

2 $$ itpeit=\alpha 1D1+\alpha 2D2+...+\alpha NDN+\gamma 1W1+\gamma 2W2+...+\gamma TWT+\beta 1 hlptit\hbox{'}+.\varepsilon it $$

Then, the model was determined within control variables. The results of likelihood and Hausman tests were the same as Model 1, that is, fixed effects with both individual and time were chosen. Model 2 could be written as.

3 $$ otpeit=\alpha 1D1+\alpha 2D2+...+\alpha NDN+\gamma 1W1+\gamma 2W2+...+\gamma TWT+\beta 1 hlptit\hbox{'}+\beta 2 hlpnit\hbox{'}+\beta 3 DPIit\hbox{'}+\varepsilon it, $$

4 $$ itpeit=\alpha 1D1+\alpha 2D2+...+\alpha NDN+\gamma 1W1+\gamma 2W2+...+\gamma TWT+\beta 1 hlptit\hbox{'}+\beta 2 hlpnit\hbox{'}+\beta 3 DPIit\hbox{'}+\varepsilon it. $$

Table 1 presents the variable description.

**Table 1:** Variable description

Variables	Types	Descriptions
*otpeit*	Dependent variable	Per visit of hospital outpatient PE
*itpe*_*it*_	Dependent variable	Per capita of hospital inpatient PE
*hlpt*_*it*_	Independent variable	Number of hospital pharmacists per 1 million of population
*hlpn*_*it*_	Control variable	Number of hospital physicians per 1 million of population
*DPI*_*it*_	Control variable	Drug price index
*α*i	Individual intercept coefficient	
*γt*	Time intercept coefficient	
*Di*	Dummy variable	$$ {D}_i=\Big\{{\displaystyle \begin{array}{cc}1,& If\kern0.17em belongs\;i\;i=1,2,....,N\\ {}0, Else& \end{array}} $$
*Wt*	Dummy variable	$$ {W}_t=\Big\{{\displaystyle \begin{array}{cc}1,& If\kern0.17em belongs\;t,t=1,2,....,N\\ {}0, Else& \end{array}} $$
*Ε*_*it*_	Error term	

## Results

### Brief descriptive statistics

**Fig. 1:**
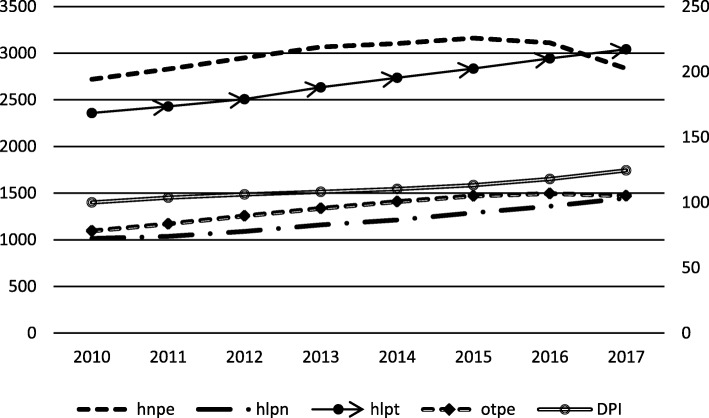
Time trends of a variable’s average

Figure 1 shows the time trends of the average of five variables. OTPE had an upward trend from 2010 to 2016 but had a downward trend in 2017. ITPE also showed an increasing trend from 2010 to 2015 but exhibited a decreasing trend from 2016 to 2017. HLPT, HLPN and DPI increased per year. The results revealed that a series of policies for hospital PE control successfully reduced PE but failed to restrain price increase.

Relationship between the number of hospital pharmacists and hospital PE.

As shown in Table 2, the number of hospital pharmacists in Model 1 was significantly (*p* < 0.01 and *p* < 0.01) and negatively (β_1_ = − 0.0893 and − 4.924) associated with OTPE and ITPE. R^2^ of 0.988 and 0.978, which were almost equal to 1, indicated the goodness-of-fit reached a high degree. A unit increase in HLPT led to a decrease of ¥0.0893 ($0.0127) OTPE and ¥4.924 ($0.700) ITPE. In Model 2, the relationship between the number of hospital pharmacists and hospital PE was still significant (*p* < 0.01 and *p* < 0.01) and negative (β_1_ = − 0.141 and β_1_ = − 4.771). R^2^ also suggested the goodness-of-fit reached a high degree. The result illustrated in the circumstance of controlling the number of physicians and an increase in drug price, a unit increase in HLPT led to a decrease of ¥0.141 ($0.0200) OTPE and ¥4.771 ($0.678) ITPE. HLPN was significantly (*p* < 0.01) and positively (β_2_ = 0.109) associated with OTPE, whereas the relationship with ITPE was not significant (*p* > 0.1). The result illustrated a unit increase in HLPN accelerated the ¥0.109 ($0.0155) increase in OTPE. DPI was significantly (*p* < 0.01 and *p* < 0.05) and positively (β_3_ = 0.206 and β_3_ = 9.636) associated with OTPE and ITPE. Furthermore, a 1% increase in DPI on the basis of 2010 accelerated a ¥0.206 ($0.0293) increase in OTPE and a ¥9.636 ($1.370) increase in ITPE.

**Table 2:** Results of fixed effects with both individual and time

Variables	Model 1 (no control variables)		Model 2 (within control variables)	
Parameters	OTPE	ITPE	OTPE	ITPE
β1	−0.0893 (0.036)**	−4.924 (1.31)***	−0.141 (0.048)***	−4.771 (1.05)***
β2			0.0183 (0.0047)***	0.109 (0.26)
β3			0.206 (0.070)***	9.636 (3.74)**
C	112.714	3916.778	77.944	2691.983
R^2^	0.987	0.978	0.988	0.978
N	248	248	248	248
F	432.63	239.10	424.83	232.93
Prob(F-statistic)	0	0	0	0

NOTE: standard error is in the parentheses, ***p < 0.01, **p < 0.05, *p < 0.10

### Further explanation of the regression results

First of all, in accordance with the total population of China in 2017, i.e. 1.39 billion, a unit increase in HLPT was equivalent to an increase of 1390 hospital pharmacists.

While the hospital’s outpatient visits in 2017 were 3,363,024,294, and the number of inpatients was 189,154,280, so an increase of 1390 hospital pharmacists reduced ¥474 million ($67.4 million) hospital outpatient PE and ¥902 million ($128 million) hospital inpatient PE. The total reduction was ¥1.377 billion ($196 million) hospital PE.

Conclusively, an increase of one hospital pharmacist could save almost ¥1 million ($142 thousands) hospital PE each year.

The results were in view of the current situation of pharmaceutical expenditure and the development of clinical pharmacy in China.

## Discussion

We analysed the relationship between the number of hospital pharmacists and hospital PE by constructing an 8-year provincial panel dataset. Several points should be further discussed.

The significant increase in hospital pharmacists was related to a decrease in hospital PE. After the number of physicians and DPI increased, the effect did not remarkably change. In particular, an increase in hospital pharmacist per 1 million of population could decrease ¥0.141 ($0.0200) OTPE and ¥4.771 ($0.678) ITPE. Previous micro-perspective studies in China have also confirmed that clinical pharmacists can reduce PE [31–33]. Jiang et al. [31] found that the full-time clinical pharmacy services in ICU decrease PE 40.07USD for an average of every patient by comparing pre- and post-intervention phase. Jiang et al. [32] showed that pharmacists adjust doses for critically ill patients who receive continuous renal replacement therapy, possibly saving 1225.47 USD per patient on average. Shen et al. [33] found that the intervention of clinical pharmacists on antibiotic use for inpatients with respiratory tract infections can averagely save 111.9 USD for every patient. Thus, statistically our result is consistent with the micro-perspective study in China.

However, our results of PE decreasing are minimal comparing with the above micro-perspective researches. The reasons of this phenomenon can be explained from two aspects, one is the policy background, the second is our study method. Firstly, the differences indicated that clinical pharmacists in China cannot provide comprehensive and professional pharmaceutical care [26]. From external environment, governments and hospital administration do not regulate concrete assignment for clinical pharmacists, and the guideline of pharmaceutical care is not released, so the work scope is unclear [27]. For the clinical pharmacists themselves, they cannot give authoritative advice to physicians because of the deficiency of professional knowledge. Some pharmacists are worried of bearing responsibility, so they seem reluctant to be involved in drug therapy [27]. Considering the theory of planned behaviour, He et al. [34] studied the psychosocial predictors of Chinese hospital pharmacists’ intention to provide clinical pharmacy services. They found clinical pharmacists prefer providing auxiliary clinical pharmacy services (CPSs) to core CPSs. Three main themes affecting the predictors of core CPSs in turn are the intention to provide auxiliary CPSs, attitude and subjective norm. These situations indicate the external support from government and hospital administrators, and the cognition and sense of responsibility from clinical pharmacists are crucial to fully offer pharmaceutical care and effectively reduce PE. Secondly, the differences are also related to our research method, that is, a macro panel model. On the one hand, owing to using the real world data, the results had some differences compared with the micro-perspective researches which were conducted on small groups, so from micro perspective, per capita PE was not much reduced. However, from a macro-perspective, increasing one hospital pharmacist per 1 million people could save a total of ¥1.377 billion ($196 million) hospital PE in 2017, so the total hospital PE decline was considerable. On the other hand, micro-perspective studies have mainly focused on the concrete interventions implemented by pharmacists [10], but our study concentrated on the number of pharmacists, which illustrated human resource allocation. Therefore, practical meaning may have differences.

Comparing the reduction in outpatient with inpatient PE, the coefficient of 4.771 ITPE obviously exceeded 0.141 OTPE, which had a difference of 33.84 times. The reason may rely on clinical pharmacists who tend to offer more services to inpatients [25]. They can provide comprehensive drug therapy, including the observation of pharmacokinetic parameters, timely adjustment of medication regimens, review of prescriptions and working as a part of a multidiscipline, because they have more contact with inpatients and grasp more information [35]. Furthermore, clinical pharmacists can provide medication counseling for patients and their families [36], so clinical pharmacists may save more PE for inpatients.

Physicians may increase hospital outpatient PE. Many papers [22, 27, 37] have reported that physicians likely receive kickbacks from healthcare companies when they prescribe target drugs in China. Some physicians’ bonuses are based on prescriptions [21]. Driven by profit, physicians choose to prescribe more drugs or expensive drugs for outpatients [38], causing an increase in hospital outpatient PE. Therefore, using the experience of other countries as a reference, clinical pharmacists should have the right to conduct a medication review to restrain physicians [4, 26].

We could draw the conclusion it was statistically significant that hospital pharmacists had an effect on reducing PE, although the effect is minimal in current situation. External supports such as governments specifying pharmacist’s responsibility [26], universities developing education on clinical pharmacy [39], and hospitals enhancing training on pharmaceutical care [40] are urgently needed. Moreover, clinical pharmacists need the consideration and understanding from physicians, patients and society [26]. The Chinese PLA General Hospital constructed a work model and management system for clinical pharmacists by making a standard work flow chart and a series of standard registration forms, pharmaceutical and practical manuals and clinical pharmacy information support system [40]. Therefore, we need to accelerate the development of talents of clinical pharmacy, increase the number of clinical pharmacists and facilitate them to play the full part of pharmaceutical care, thereby effectively solving the problem of controlling PE in China’s health reform [5, 40].

### Strengths and limitations

This study was the first to use the panel data model of fixed effects and analyse the relationship between the number of hospital pharmacists and PE in China, but this study still had the following strengths and limitations:

#### Theoretical significance

Different from previous micro-perspective studies confirmed the PE-reducing effect of clinical pharmacists by clinical intervention trials, this study built a macro model of the number of hospital pharmacists and PE, which reflects the current situation of clinical pharmacy development in China.

#### Practical significance

The results of this study revealed that we should completely focus on the positive effect of hospital pharmacists in reducing PE and expanding the growth of hospital pharmacists. The coefficient of two variables was low, indicating that clinical pharmacists might not be well organised in their roles. Therefore, the quality of pharmaceutical care should be improved, and clinical pharmacists should be encouraged to participate in drug therapy, such as medication reconciliation through a series of regulations.

#### Limitations

This study focused on hospital pharmacists. Some micro-level studies have shown that community pharmacists’ intervention in patient care can also reduce PE [41, 42]. For example, Sanyal et al. [41] found that community pharmacists prescribing antibiotic treatment regimens for patients with uncomplicated urinary tract infection can save more medication costs than physicians. Natasha et al. [42] observed that the medication review of community pharmacists can save $107 for every Medicaid beneficiary. However, Chinese community scarcely employed clinical pharmacist owing to the preliminary stage of pharmaceutical care. In addition, no authoritative statistics are available [43]. With the development of pharmaceutical care in the community, the effect of community pharmacists should be further investigated.

## Conclusion

This study demonstrated that the increase of hospital pharmacists could help to decline OTPE and ITPE. The results indicated that we should give more support to clinical pharmacy and increase the number of clinical pharmacists with high professional accomplishment. So that we can slow down the sustainable growth of PE and reduce improperly medication use without decreasing therapeutic effect.

## Supplementary information

**Additional file 1.** The data is constituted of two sheets. Sheet 1 named ‘panel data for analysis’ which included all variables using for empirical analysis. Sheet 2 named ‘original data’ which included the original data abstracted from yearbooks and website of the National Bureau of Statistics of China. The first two rows were individual and time terms then came to the value of variables

## Data Availability

The dataset supporting the results is included within the article and Additional file 1. The data of hospital pharmacists, physicians and drug costs were extracted from China Health Statistics Yearbook of 2011 to 2018. Population data were extracted from China Statistics Yearbook of 2018. The data of DPI were extracted from the website of the National Bureau of Statistics of China. All public access to the databases is open. The links/references are as follows: “National Health Commission Of China. China Health Statistics Yearbook. Beijing: Peking Union Medical College Press; 2018. National Bureau of Statistics Of China. China Statistics Yearbook. Beijing: China Statistics Press; 2018. Retail Price Index of Drugs and Medical Care Products. National Bureau of Statistics. Available at http://data.stats.gov.cn/easyquery.htm?cn=E0103&zb=A0904&reg=110000&sj=2017.”

## Abbreviations

CPSs: Clinical pharmacy services
DPI: Drug price index
HE: Health expenditure
HLPN: Number of hospital physicians per 1 million of population
HLPT: Number of hospital pharmacists per 1 million of population
ITPE: Per capita of hospital inpatient pharmaceutical expenditure
IV: Intravenous
OTPE: Per visit of hospital outpatient pharmaceutical expenditure
PE: Pharmaceutical expenditure
